# On the Merits, Real and Comparative, of Amputations in the Lower Third of the Leg, with Suggestions as to the Prospective Value of Periosteal Flaps

**Published:** 1875-04-15

**Authors:** 


### ON THE MERITS, REAL AND COMPARATIVE, OF AMPUTATIONS IN THE LOWER THIRD OF THE LEG, WITH SUGGESTIONS AS TO THE PROSPECTIVE VALUE OF PERIOSTEAL FLAPS.

*From the New York Medical Journal, April, 1875.*

DR. Stephen Smith read the paper of the evening on the above subject of which the following is a brief synopsis:

Ambrose Pare, three hundred years ago, related a case of a man who had his foot shot off, and, after it had healed, had the leg amputated five finger-breadths below the knee, on account of the burden it proved to be in walking.

Pare, in commenting on this subject, says: "We must do otherwise if any such thing happens in the arm;"—and thus shows how accurately he appreciated a most important principle in modern surgery, which is as follows: *The special function of the limb should be considered, and as far as possible preserved in selecting the point and method of operation.* Unfortunately, however, if a score of limbs are examined, few, if any, will show that the surgeon was governed in the point of election of his amputation by the special functions of the limb, and the facility of adapting to it artificial appliances. It is most important to the patient that this be well considered, as it may determine whether he is to be a pauper for the rest of his days, or a self-supporting citizen. And this may best be arranged by a consultation of the operating and mechanical surgeons.

There has been much difference of opinion in regard to the relative advantages of amputation of the leg below the knee, and at the lower third, in regard to its usefulness to the patient for the adaptation of an artificial limb. When patients were only able to obtain the wooden "pin and bucket," it was an advantage to have a short stump; but with the improvement in artificial appliances, it was found that a leg which had been amputated at its lower third could have applied to it an apparatus which would enable the patient to walk with great facility.

In our own day the principles which should govern surgeons in the election of the site and method of amputation of the leg are vague and not defined; and the results by no means sustain Pare's proposition in regard to the special function of the limb. One of the chief reasons for the diversity of surgical opinion in regard to amputations is, that not enough attention is given to the consideration of the mechanical aids which can be adapted to substitute the natural extremity; whereas, at the present time, artificial limbs have reached to such a point of perfection, that in the lower extremities they may be said to completely restore function and symmetry when the stumps admit of their proper application; and, moreover, they are sufficiently cheap to be within the unaided or aided means of all. As regards amputation of the leg, the two governing rules are: 1. Safety to the life of the patient; 2. Serviceableness of the stump. As regards the first, the chances for the life of the patient are doubled by the low operation; and, as regards the second, it depends on the point and method of the amputation.

To render a stump useful, it is necessary that it be absolutely free from tenderness, and the principal cause of tenderness is a thin, imperfect cicatrix attached to the bone. It is usually the result of sloughing, imperfect, or retracted flaps, which in themselves are produced by an operation yielding too thin coverings to the bone.

Tenderness may best be avoided by having the cicatrix situated anteriorly or posteriorly to the end of the stump, and the bone covered by a thick, fleshy mass, as practiced by Teale, of Leeds, or Lee, of London.

An important question arises here as to whether it is necessary for patients to bear their weight on the end of the stump, and the expert mechanicians answer, "Yes." Dr. Hudson, of this city, one of the best students of the mechanism of artificial appliances in this country, gives it as his opinion that a stump that will take the weight of the body is useful, whatever the method of support may be. And, if this is so, an important question that arises is, what part the periosteum may perform in this respect, when it is employed in covering the end of the bone. Since 1814 periosteal flaps have at different times been suggested, and, during the winter of 1862–'63, Assistant Surgeon G. M. McGill, U. S. A., practiced the use of these flaps to nourish the bone. His method was, to raise a section of the periosteum from the anterior surface of the bone sufficient in area to cover the cut extremity of the bone. The stumps are described as being well rounded, and capable of bearing pressure. The advantages which have been attributed to this method are: 1. That the cicatrix does not become attached to the bone. 2. That osteo-myelitis is prevented. 3. That the stump presents a rounded form and is firm.

The disadvantages are: 1. That the periosteum being poorly nourished, is liable to slough. 2. If it does become attached, osteophytes may develop, which will destroy the stump.

---

PHAGEDENIC CHANCROID.—The patient came into hospital suffering from a sloughing chancroid. It began as a soft chancre on the prepuce. After taking on a phagedenic character it involved and destroyed the penis, and then extended down and spread over the thigh. All treatment proved useless to stop its progress, the actual cautery proving absolutely of no avail.

